# Plague epizootic cycles in Central Asia

**DOI:** 10.1098/rsbl.2014.0302

**Published:** 2014-06

**Authors:** Jonas Reijniers, Mike Begon, Vladimir S. Ageyev, Herwig Leirs

**Affiliations:** 1 Department of Biology, University of Antwerp, Antwerp, Belgium; 2 Institute of Integrative Biology, University of Liverpool, Liverpool, UK; 3 Kazakh Scientific Centre for Quarantine and Zoonotic Diseases, Almaty, Republic of Kazakhstan

**Keywords:** plague, abundance threshold, flea, gerbil, vector-borne disease, predator–prey cycle

## Abstract

Infection thresholds, widely used in disease epidemiology, may operate on host abundance and, if present, on vector abundance. For wildlife populations, host and vector abundances often vary greatly across years and consequently the threshold may be crossed regularly, both up- and downward. Moreover, vector and host abundances may be interdependent, which may affect the infection dynamics. Theory predicts that if the relevant abundance, or combination of abundances, is above the threshold, then the infection is able to spread; if not, it is bound to fade out. In practice, though, the observed level of infection may depend more on past than on current abundances. Here, we study the temporal dynamics of plague (*Yersinia pestis* infection), its vector (flea) and its host (great gerbil) in the PreBalkhash region in Kazakhstan. We describe how host and vector abundances interact over time and how this interaction drives the dynamics of the system around the infection threshold, consequently affecting the proportion of plague-infected sectors. We also explore the importance of the interplay between biological and detectability delays in generating the observed dynamics.

## 1. Introduction

The idea of a host abundance threshold or critical community size [1], which has to be exceeded for an infectious agent to invade and/or persist in a host population, is a core concept in epidemiology, but has only rarely been demonstrated in wildlife populations [2], despite its importance commonly being emphasized [3]. The plague system in Kazakhstan has proved valuable in demonstrating key aspects of abundance thresholds in wildlife populations. Plague (*Yersinia pestis* infection) [4] circulates there, transmitted by fleas (mainly *Xenopsylla* spp.), in populations of great gerbils (*Rhombomys opimus*) that live as extended family groups in spatially discrete burrow systems distributed across the landscape. Davis *et al*. [5] demonstrated the existence of an abundance threshold below which the infection was not detected, abundance being measured by occupancy (O), the proportion of burrow systems occupied by gerbils. A subsequent paper [6] provided support for this being a percolation threshold: as gerbil abundance declines, occupancy can decrease to levels where plague can no longer percolate, because the occupied burrows are too sparse given the distances gerbils regularly travel. However, the occupancy threshold still gave rise to many ‘false positives’: sampled sectors for which theory predicted the potential presence of plague, yet where no plague was isolated. As fleas are the vectors for transmission, it is likely that plague cannot percolate if the flea burden (F), the average number of fleas carried per gerbil, is too low. Supporting this, model simulations combining F and the density of occupied burrows to generate a hyperbolic threshold proved superior to the earlier model (ΔAIC = 13.4), reducing the number of false positives when applied to field data [7].

In these analyses, the threshold was not assessed solely from field data from the current year, but as a (weighted) average incorporating data from previous years. For the gerbil threshold model, the prediction was best if the two preceding years were included. Indeed, the data with a 2-year delay carried greater weight than those with a 1-year delay. A similar observation was made when the flea burden was incorporated: the best model fit was obtained when both F and O were averaged over the two preceding years, now conflated with the current year. As discussed by Davis *et al.* [5], however, what accounts for this relation between epizootics and past rather than current abundance of gerbils and fleas has not been clear.

One possible explanation relates to plague monitoring having limited sensitivity. Given the limited number of gerbils tested, thresholds will be associated with detectable presence rather than presence *per se*. Note also that the infection threshold dictates the growth or decline of the infection, rather than its presence or absence. Thus, there may be a time delay between a host (or vector) abundance exceeding an infection threshold and the infection being detected (or between its falling below the infection threshold and the infection going unnoted) simply because it takes time for the infection prevalence to rise above (or fall below) the detection threshold. Such patterns are most likely in natural populations, which may fluctuate markedly on short time scales.

In addition, though, in systems with more than one functional host, delays may arise for biological reasons. For example, in the gerbil–flea–plague system, the abundance of the fleas—largely specific to the great gerbil [8]—is itself likely to depend on gerbil abundance following a population growth rate delay; and the abundance of fleas, as predators of the gerbils, may also affect gerbil abundance, again following a delay, completing a predator–prey cycle. Given the importance of abundance thresholds in epidemiology, insights into the way detection and biological factors may combine and contribute to threshold-response delays in natural populations will be valuable. To gain such insights, in this paper we investigate the temporal dynamics of plague, gerbils and flea burden in the PreBalkhash focus.

## 2. Material and methods

As in previous analyses, we use the long-term plague surveillance data collected in the PreBalkhash desert in Kazakhstan. Each spring and autumn between 1949 and 1995, the proportion of burrows inhabited was estimated at a variable number of locations by the Anti-Plague authorities. Gerbils and (from 1975 onwards) fleas were trapped and tested for *Y. pestis* infection; samples were pooled at the 10 × 10 km sector level [7]. Plague was tested for by plating tissue samples (gerbils) or pools of fleas on Hottinger's agar containing 1% haemolyzed sheep erythrocytes.

The ability of plague to percolate depends on the connectivity of the local burrow network, which, in an earlier analysis, was shown to depend both on O and F [7]. Moreover, both O and F have been shown to vary (separately) synchronously over a large area [7]. Consequently, we can describe the status of the whole focus with a single two-dimensional state vector (O, F), merging the vector and host data at this larger, whole-focus scale. Doing so allows us to inspect the pattern in the *annual* dynamics of the state vector and its effect on the plague dynamics. This was not possible in previous analyses, which inspected F and O at the local (sector) scale, first because not every sector was sampled every year, but also because there was too much noise on F and O at the sector level.

In this analysis, plague dynamics will also be studied at the whole-focus scale as the proportion (P) of monitored sectors that were infected (‘plague prevalence’ at the whole-focus scale). This can be directly related to the probability of plague invading locally, which was simulated in a previous analysis [7] to produce the hyperbolic threshold curve.

## 3. Results

**Figure 1.**
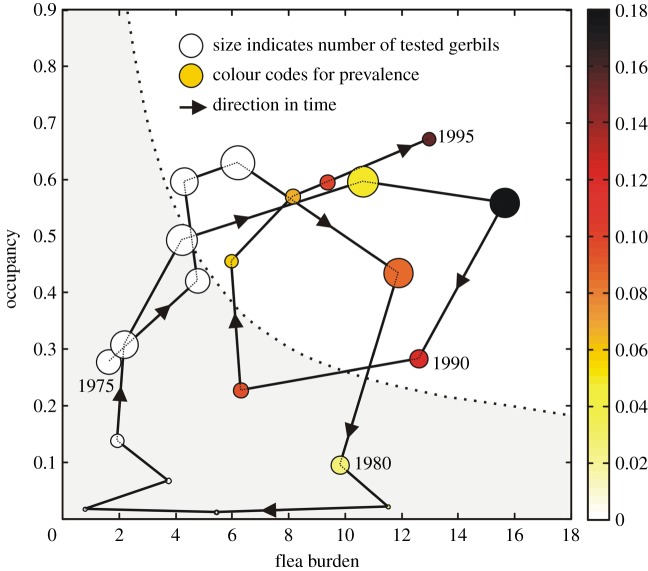
Vector–host–pathogen dynamics in the PreBalkhash region. Occupancy is plotted as function of flea burden, yearly from 1975 onwards until 1995. Flea burden and occupancy are averaged over all sampled sectors. The colour codes for the proportion of sectors that tested positive and the area of every dot is proportional to the number of gerbils tested in that year (for reference: 30 673 gerbils in 1978). Prevalence in 1981 was 0.03; in 1982 and 1983 it was zero. The dotted line corresponds to the threshold curve, derived in Reijniers *et al.* [7]. (Online version in colour.)

**Figure 2.**
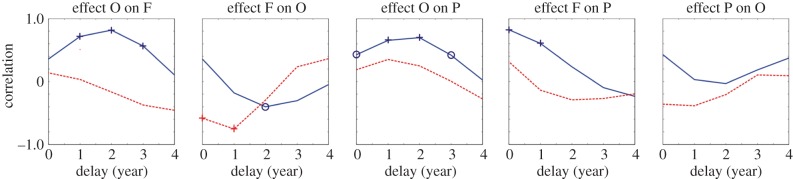
Correlations of the different longitudinal datasets (F, O, P) that make up the vector–host–pathogen cycles shown in figure 1. Solid line: pairwise correlations corr{A(*t*), B(*t* + delay)} with delays ranging between 0 and 4 years. Dotted line: the same but now between factor A and the growth rate of B, i.e. corr{A(*t*), [B(*t* + delay)−B(*t* + delay − 1)]/B(*t* + delay − 1)}. Symbols ‘+’ and ‘o’ mark significance levels: *p* ≤ 0.05 and *p* ≤ 0.1, respectively. (Online version in colour.)

In figure 1, occupancy is plotted as a function of flea burden, yearly, from 1975 until 1995. The state vector moves in a clockwise circular motion in F–O space. During the 20 years of data, we observe 2.5 of these cycles. This suggests that F ‘follows’ O, lagging behind by approximately 2 years. This is confirmed by Pearson correlation coefficients at different lags, derived for the association of O with F and with the growth rate in F, F′ (figure 2; Spearman correlation results similar).

In the simplest (Lotka–Volterra type) predator–prey system, the fleas and gerbils would be fully coupled. Not only would the fleas increase (following a delay) when gerbil abundance was high, but the gerbils, as prey, would also decrease (following another delay) when flea burden was high. This, too, is confirmed, with a similar lag (figure 2), though the effect is weaker. Note, though, that flea burden may be acting as a proxy for the abundances of a range of gerbil ‘predators’ with which flea burden is positively correlated, rather than the fleas alone having a direct effect on gerbil abundance.

The looping of the state vector in F–O space is associated in turn with plague dynamics. This is indicated by the colours in figure 1 that code for the proportion of infected sectors and is confirmed by the correlation coefficients (figure 2). Plague prevalence follows burrow occupancy with a lag of approximately 2 years and flea burden with a lag of roughly 1 year. Plague itself had no detectable effect on gerbil abundance.

To further interpret plague prevalence, we have plotted the combined gerbil–flea threshold curve (figure 1) as deduced from model simulations and fitted to field data [7] (with the assumption that for all sectors the burrow density equals the average density in the focus of 3 burrows per hectare (estimated by the Anti-Plague authorities, personal communication 2013)). The threshold curve can be interpreted as a phase transition line, separating network geometries that support plague from those that do not. Thus, when the state vector rises above the threshold (upper left) this is followed by an increase in plague prevalence. Prevalence peaks when the state vector is furthest from the threshold curve and decreases and sometimes disappears when, a few years later, the state vector again falls below the threshold (lower right).

Hence, the longitudinal whole-focus prevalence data seem to reaffirm the threshold curve. However, delays are apparent. In 1977, the threshold curve was exceeded, but it was not until the third year above the threshold, 1979, that plague was detected. Similarly, in 1980, the state vector fell and remained below the threshold, yet prevalence did not fall to zero until 1982. Moreover, plague does not necessarily disappear when the state vector loops below the threshold. For example, during the second cycle, the state vector was below the threshold for only 1 year, 1991, and plague was still detected.

## 4. Discussion

These results explicitly support the importance of interplay between biological and detectability delays around abundance thresholds and are the first to do so. For plague to spread, burrow occupancy must be sufficiently high *and* gerbils must be carrying sufficient numbers of fleas [7]. But flea burden follows occupancy with a 2-year delay, so this may account for the behaviour of the system in the late 1970s and again in the 1980s (figure 1), when occupancy exceeded its threshold but flea burden did not, and plague was not detected until 1979 and 1988, respectively. It may also explain why the gerbil threshold model [5] relied on past burrow occupancy levels (especially with a 2-year delay), rather than on more recent levels. But even in this case, when flea burden is taken into account, there are several examples where the presence or absence of plague is wrongly aligned with the position of the system above or below the threshold: 1977, 1978, 1980, 1981 and 1991. These are likely to be due to the limited responsiveness of detectable plague prevalence to an increase in gerbil and flea abundances: if the threshold is exceeded, it seems that the infection needs time to grow to detectable prevalence levels. Similarly, if the state vector falls below the threshold, it takes time for plague to fade out to levels that go undetected. This may explain why in the joint threshold model, too, the previous 2 years had to be taken into account [7].

A 2-year lag in detectability may seem large, but we note, first, that breaching a threshold precipitates a growth in infection, and that growth is exponential and likely to be from a low starting point. Compounding this, the surveillance programme is itself insensitive. Plague prevalence in gerbils is always low (maximum recorded prevalence in gerbils was 0.06 [5]), and although the total number of gerbils tested was often large (varying dramatically between 85 and 30 673, see dot sizes in figure 1), these are caught in a large number of sectors, such that, on average, only 80 gerbils approximately are trapped in each sector, which will limit sensitivity, especially if the initial appearance of plague is local, as it is likely to be.

Merging the data into one single O–F state vector comes at a price, though, as it averages out the spatial heterogeneity in both occupancy and flea burden, which may also explain deviations from the phase diagram. For example, when the state vector is below the threshold, plague may still be detected because (the state vectors of) some sectors are (still) above the threshold. Nonetheless, it seems clear overall that biological and detectability effects combine to account for the delays previously observed in the gerbil and gerbil–flea threshold models [5,7]. The results emphasize, too, that the seemingly disorganized distribution of observations in either O or O–F space, reported in these previous threshold studies, are in fact structured time series reflecting the underlying dynamics of the system.

## Supplementary Material

Data used in the analyses/figures

## Supplementary Material

Raw data

## Data accessibility

The dataset supporting this article has been uploaded as part of the electronic supplementary material.
